# Differences in Spatial Physical Activity Patterns between Weekdays and Weekends in Primary School Children: A Cross-Sectional Study Using Accelerometry and Global Positioning System

**DOI:** 10.3390/sports4030036

**Published:** 2016-06-27

**Authors:** Rahel Bürgi, Eling D. de Bruin

**Affiliations:** Department of Health Sciences and Technology, Institute of Human Movement Sciences and Sport, ETH Zürich, Leopold-Ruzicka-Weg 4, Zurich 8093, Switzerland; eling.debruin@hest.ethz.ch

**Keywords:** physical activity, accelerometer, GPS, children, location, weekend, weekday

## Abstract

Targeting the weekend to promote physical activity (PA) in children seems to be promising given that they tend to be less physically active and, particularly, as the age-related decline in PA is more marked during weekends. Considering the ambiguity of why children are not able to maintain their PA level on weekends, the aim of the present study was to objectively investigate differences in children’s spatial PA patterns between week and weekend days using the combination of Global Positioning System (GPS) and accelerometry. Seventy-four second graders (aged 7–9 years) and 98 sixth graders (aged 11–14 years) wore an accelerometer and GPS sensor for seven consecutive days to determine where children spend time and engage in PA. Time-matched accelerometer and GPS data was mapped with a geographic information system and multilevel analyses accounting for the hierarchical structure of the data were conducted. Differences between weekdays and weekends regarding the total time spent and the absolute and relative level of PA in various settings were found in both age groups. The findings support previous research pointing to the importance of targeting weekend PA, especially when children grow older. Future interventions should encourage children to use outdoor spaces more frequently on weekends, rather than stay at home, and to commute actively to destinations other than school.

## 1. Introduction

Regular physical activity (PA) in children is associated with many physical and psychological health benefits, and there is a widespread belief that PA contributes to children’s social development [1,2,3]. Evidence is present that PA performed during childhood may also contribute to an active lifestyle in adulthood [4,5]. Current international public health guidelines for children recommend at least one hour of moderate-vigorous PA (MVPA) per day [6]. However, only a small percentage of school-aged children achieve these recommendations [7,8]. In Switzerland, only one in five boys and just only one in ten girls aged 11 are adequately physically active according to these PA recommendations [8]. In light of this evidence, the promotion of an active and healthy lifestyle during childhood and adolescence should be an urgent public health priority.

In order to develop effective evidence-based public health interventions, it is essential to have a clear understanding of children’s PA patterns and its influencing factors [9]. A large number of studies have been conducted in the last ten years to assess patterns and correlates of children’s PA, in which gender, age, socioeconomic status, and parental and peer influences were among the most investigated correlates [10]. However, since previous studies mostly rely on subjective assessment methods that are subject to recall bias, knowledge on children’s PA is still incomplete [11]. Using objective high-frequency accelerometry, which facilitates the analysis of temporal correlates, previous studies were able to find notable differences in PA level between weekdays and weekends [12,13,14]. Given that school-aged children generally accumulated more health-enhancing PA on weekdays than on weekends, Brooke and colleagues [12] pointed to the importance of targeting weekend PA for future interventions. Moreover, different studies showed that the well-investigated decline in MVPA across age groups between childhood and adolescence is more marked on weekends than weekdays [14,15,16], which also supports interventions focusing on PA maintenance on weekend days.

Although there is growing evidence about the discrepancy in PA level between weekdays and weekends, it is still unclear why children are not able to maintain their weekday PA level on weekends. It is suggested that the low accumulation of MVPA during weekends can partly be explained by the absence of the structured school environment, a greater television viewing time, or a more significant familial influence [17,18,19]. The longer sleep duration at weekends, which limits waking hours available for recreational pursuits and PA, may also be an influential factor [20]. To shed light on this issue, future studies encompassing multidimensional correlates have to investigate differences in activity patterns between weekdays and weekends in more detail [12,21].

Along with the increased use of ecological models in public health, also the environment in which PA takes place has become a stronger focus of attention [22]. Primarily, environmental interventions and changes are thought to have a high potential for sustained impact on populations, especially on those groups that are hard to reach with PA promotion programs [23]. A growing body of research emphasises that environmental factors may have a crucial influence on children’s PA patterns [24], but this influence seems to be highly context-specific [25,26]. Therefore, to guide more targeted PA promotion programs, knowledge on differences in context-specific PA between weekdays and weekends may be a promising step.

Recent technological advances have led to the advent of Global Positioning System (GPS) in PA related studies [27,28]. The development of lightweight, accurate, and affordable GPS devices has opened up new possibilities to investigate how people move within their neighbourhood [27,29]. When combining these devices with accelerometers, objectively measured PA can be linked to the location in which the activity takes place, thereby providing a spatial context of PA. Given that the combination of GPS and accelerometry provides objective, reliable, and accurate measurements of context-specific PA [30,31], studies using this objective approach are increasing in numbers and new evidence about context-specific activity patterns is being generated. A review by McCrorie and colleagues [28], which summarises the current literature about using the combination of GPS and accelerometry in children and adolescents, has indicated that objectively measured environmental characteristics can significantly influence children’s PA levels. Moreover, they showed that roads, school grounds, and the home environment are particularly important for both total PA and MVPA [28]. However, nearly none of the studies using GPS and accelerometry examined if there were any differences in the context-specific PA patterns between week and weekend days in primary school children. Klinker and colleagues [32] provided baseline results on both the use of different domains and the time spent in these domains separately for week and weekend days. However, neither did they conduct formal statistical tests of differences between weekdays and weekends nor did they investigate whether there are differences between these time segments relating to the accumulation of MVPA.

Using the combination of GPS and accelerometry, the aim of the current study therefore was to assess objectively the spatial PA patterns during weekdays and weekends in school-aged children living in an urban area in Switzerland. In particular, the goal was to investigate if there were any significant differences in context-specific PA between week and weekend days in both second- and sixth-grade children. In addition to existing literature, we aimed to consider not only the use of and total time spent in different settings, but also the time spent in health-enhancing MVPA. Given that reporting both relative and absolute amounts of PA can provide complementary information [12,33], we used the amount of MVPA in minutes as an absolute outcome and the proportion of MVPA in % as a relative outcome to examine differences in the context-specific PA behaviour between week and weekend days.

## 2. Materials and Methods

### 2.1. Setting

To investigate spatial activity patterns of Swiss primary school children in relation to gender and socioeconomic differences, we previously conducted two cross-sectional studies in two different municipalities in the German-speaking part of Switzerland. During January and April 2013, a convenience sample of sixth-grade children participated in a first study carried out in Winterthur [33]. Winterthur is the sixth largest city in Switzerland, with a population of over 100,000 residents. One year later, between February and June 2014, a convenience sample of second-grade children participated in a second study conducted in the municipality of Zurich [34]. With a population of over 400,000 residents, Zurich is the largest city in Switzerland. Both cities represent an urban area, which is representative in relation to various demographic values (e.g., unemployment rate, foreign nationals, or social assistance rate) [35]. In both years, the same recruitment and data collection procedures were applied, which enabled us to aggregate the data for the current study to investigate differences in the spatial PA behaviour between week and weekend days in a sample of both second- and sixth-grade children.

### 2.2. Participant Recruitment

After approval of the school authorities, relevant teachers from public schools in one of the two municipalities were informed in cooperation with the local office of sports by an information letter. If a teacher agreed to take part, his class was visited by a trained study team consisting of the first author [RB] and a student research assistant during a lesson. The study team briefed the class about the study and provided all children with an information letter and an entry form for their parents. To be eligible to participate, children had to be free from any severe disability which prevented them from engaging in routine everyday PA. For every pupil in a participating class, the participation was voluntary and parental written informed consent was obtained. Ethical approval for both studies was obtained from the Ethics Committee of the ETH Zurich (EK 2012-N-62; EK 2013-N-66).

### 2.3. Data Collection and Measures

**Figure 1:**
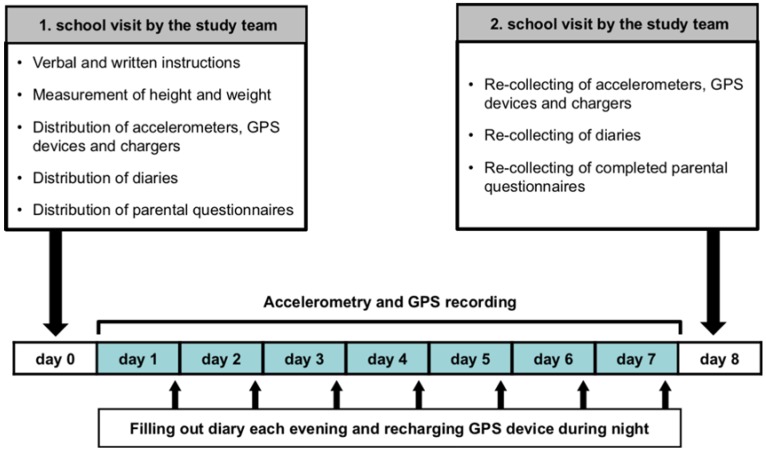
Overview on the data collection during the measurement week. GPS = global positioning system.

Data were collected in Winterthur during March and April 2013 and in Zurich during May and June 2014, respectively. It was ensured that all measurements took place within a regular school week without irregular days off from school or school excursions. One day before the start of the measurements, the study team visited the corresponding class again and fitted each registered child with an elastic belt equipped with a tri-axial accelerometer (GT3X, Actigraph, Pensacola, FL, USA) to measure PA and a GPS receiver (BT-Q1000XT, QStarz, Taipei, Taiwan) to record the geographical location (Circular Error Probability, CEP, (50%) < 3 m). Both devices offer accurate measures and are suitable for use in larger population studies with a data collection period of seven or more days [36,37]. The study team configured the devices for each child in advance setting them to record at ten second intervals with their internal clocks synchronized. This rather high sampling frequency was used to reflect children’s PA pattern accurately, which is spontaneous and intermittent in its nature [38]. All children were given a detailed verbal and written instruction on belt wear which involved wearing the belt around the waist from waking to bedtime on seven consecutive days starting the next day. Due to the limited battery life of the GPS receiver, each child was provided with a charger and was instructed to recharge the receiver during each night when asleep. Children further received a small diary in which they had to report times they woke up and went to bed and times and reasons when the belt was not worn throughout the week. To obtain information on children’s age, sex, home address, and sports club membership, a short parental questionnaire was handed out. The same instrument served to assess factors of children’s individual socio-economic status (e.g., nationality, language spoken at home, parental education and income). In addition, height and weight were measured using a stadiometer (seca 213, Seca AG, Hamburg, Germany) and a digital scale (Beurer GS 12, Beurer GmbH, Ulm, Germany) with participants wearing light indoor clothing and shoes removed. Daily meteorological data, such as mean temperatures (in °C), sum of precipitation (in mm), and the sum of sunshine duration (in min), were provided by MeteoSwiss for each measurement week [39]. One day after the last day of measurements, children had to bring all instruments back to school, where they were collected by the study team. Figure 1 gives an overview on the data collection during the measurement week.

### 2.4. Data Merging and Processing

After manually reviewing each participant’s GPS and accelerometer data file to ensure that they contained no incomplete or erroneous data, GPS and accelerometer data were matched by date and time. We used existing software (Actilife 6.5.2, Actigraph, Pensacola, FL, USA) for this process producing for each recorded GPS point a measure of activity and location. Only accelerometer data from the vertical axis was considered for further analysis since current accelerometer cut points for different activity levels are based on vertical axis counts. The processing of the matched data was performed using MATLAB R2012a (MathWorks, Natick, MA, USA) and R 3.1.2 (R Development Core Team, Vienna, Austria). Intervals with >60 min of consecutive zero activity counts were classified as non-wear time and excluded from analysis [40]. Activity records >5461 counts per 10 s were identified as outliers [41] and replaced with the mean of the previous and the following value. Based on age-appropriate cut-points [42,43], each data point was then classified as sedentary (<101 counts per minute (CPM)), light (101–2295 CPM), moderate (2296–4011 CPM) or vigorous (≥4012 CPM) activity. To remove GPS outliers from the data, we processed the data by visual observation as well as automatic identification of invalid GPS data points using extreme changes in distance (>500 m per 10 s) and invalid values of altitude, which are not possible for the respective regions.

**Figure 2:**
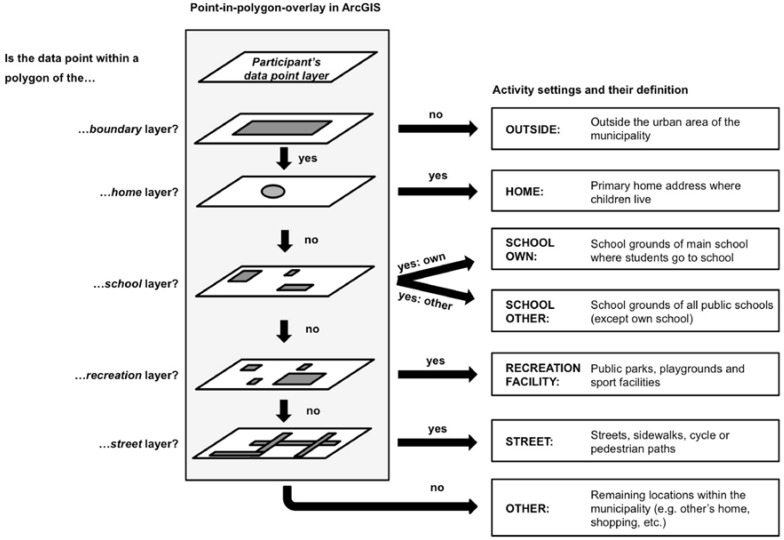
Definitions of activity settings and overview of the hierarchical assignment process in the geographic information system ArcGIS. The assignment process was conducted using the point-in-polygon-overlay, which is a spatial operation in ArcGIS. The creation of the five polygon layers *boundary*, *home*, *school*, *recreation,* and *street* was done using further geospatial data provided by the Land Surveying Office of Winterthur and the Office for Geomatics and Surveying of the City of Zurich.

The location-based categorisation of the matched data points was conducted in ArcGIS 10 (ESRI, Redlands, CA, USA). Each participant’s data points were imported into ArcGIS and plotted on their own point layer. We chose to define the seven activity settings presented in Figure 2 to assign each data point to a location. The settings used are based on similar studies in this field of research [44,45,46] and the ability to clearly assign each point to a location within ArcGIS. The assignment process was conducted in a hierarchical order using the point-in-polygon overlay, which is a spatial operation in ArcGIS. During this process described in Figure 2, each participant’s data layer was overlaid on five different polygon layers to determine which data points are contained within which polygons. The creation of the five polygon layers was done using further geospatial data (land-use data, register data, points-of-interest, and satellite imagery) provided by the Land Surveying Office of Winterthur and the Office for Geomatics and Surveying of the City of Zurich and has been described elsewhere [33]. To take the GPS measurement error into account, a buffer zone of ten meters was drawn around the school, recreation, and street polygons [30,36,47].

### 2.5. Data Analysis

Children had to provide at least one weekday and one weekend day with four full hours of matched data to be included in the analysis [33]. To improve the data quality, we only considered matched wear time data on valid days from locations in which the participant spent ≥2 min during this day.

Statistical analyses were performed using *R*. Descriptive statistics to calculate frequency distributions (number (*n*) and proportion (%)) for categorical data, mean and standard deviation (SD) for normally distributed variables, or median and interquartile ranges (IQR) for non-normally distributed data were used to describe the general characteristics of the two groups. Univariate analysis using χ^2^-test, *t*-test, or Mann-Whitney-*U*-test was performed to test differences in general characteristics between age groups.

Differences in spatial PA patterns between week and weekend days within the two age groups were examined by using the total time spent in a setting and both an absolute (amount of MVPA in min) and relative (proportion of MVPA in %) outcome measure of MVPA, as this can provide complementary information. Mean daily minutes of total time and MVPA that a participant spent in each setting were calculated as outcomes separately for week and weekend days. From these values, we further calculated the proportion of time spent in MVPA out of the total time spent in each setting separately for weekdays and weekends based on children who visited the corresponding setting. As these variables were not normally distributed, median and IQR were used to present the outcome measures stratified by age group.

For the three outcomes and separated by age group, we conducted multilevel analyses based on individual scores across days and locations to provide results on differences between week and weekend days. Models were transformed by log transformation to fulfil the model assumptions and individuals and classes were included as random effects accounting for the hierarchical structure of the data [48]. To adjust for individual differences, sex, BMI, sports club membership, total matched wear time, weather conditions (mean temperature, sum of precipitation, and sum of sunshine duration), and socioeconomic characteristics (language spoken at home, highest parental education, parental income and nationality) were included as potential confounders, as these parameters may have an influence on children’s activity patterns [49,50]. A backward elimination algorithm with Akaike’s information criterion (AIC) as a goodness-of-fit measure was applied to test the contribution of the entered predictors and to determine the final models. For each model, we used a single-step method calculating interaction contrasts to investigate differences between week and weekend days across settings. Differences were provided by the transformed and adjusted *p*-values, which were calculated from F-tests based on Sattethwaite’s or Kenward-Roger approximation.

## 3. Results

### 3.1. General Characteristics

**Figure 3:**
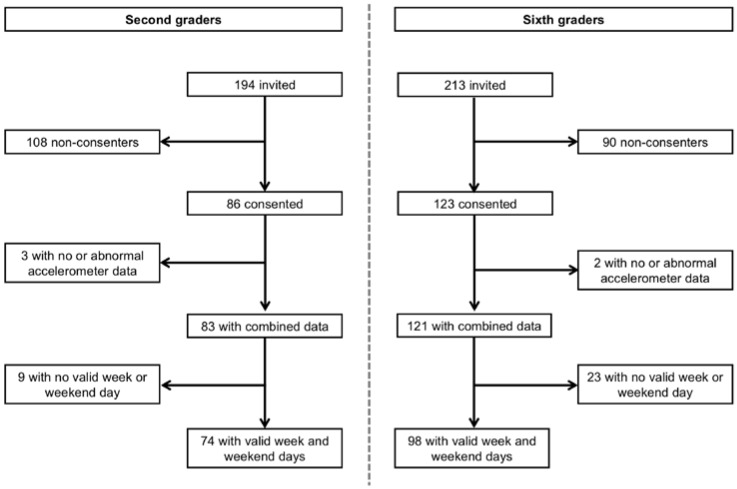
Study flow chart.

A total of 86 second graders and 123 sixth graders out of 407 invited children provided consent and wore a measurement belt during one week. One second grader and two sixth graders returned a malfunctioning accelerometer, and one second grader lost both devices during the week. One further second grader had to be excluded due to illness which prevented him from engaging in routine everyday PA. Another 32 participants did not fulfil the inclusion criteria of at least one weekday and one weekend day with four full hours of matched data. Thus, the final study population consisted of 74 second and 98 sixth graders (Figure 3). Table 1 presents the general characteristics of the study population separated by age group. Participants wore the devices during a median time of 12.8 h per day at a median of seven days. During this time, valid GPS data was available for a median of 9.7 h per day, which is equal to a median of 77.2% of the total accelerometer wear time data. In total, children reached a median of five valid weekdays and two valid weekend days. The two age groups showed similar wear time characteristics and did not differ in relation to the available amount of daily combined data.

### 3.2. Daily Minutes of Total Time Spent in Settings

#### 3.2.1. Second Graders

Table 2 provides results on the median time accumulated by the children in settings separately for weekdays and weekend days and stratified by age group. During weekdays, second graders accumulated most of their time at own school (42.4%), at home (22.5%), and on streets (14.9%), whereas home (35.2%), streets (19.2%), and other places (16.0%) were the most visited settings on weekend days (Table 2). Compared to the weekend, second-grade children recorded slightly more minutes of total matched wear time on regular school days (+42.9 min, *p* = 0.047). In nearly all settings, except for streets, we further found significant differences in daily minutes of total time between week and weekend days: While second graders spent significantly more time at both their own school and other schools on weekdays (+244.6 min, *p* < 0.001; resp. +19.6 min, *p* = 0.02), home (+38.3 min, *p* < 0.001), recreational facilities (+3.9 min, *p* < 0.001), other places (+7.4 min, *p* = 0.032), and the area outside their home city (+5.7 min, *p* < 0.001) were significantly more frequently used on weekends.

#### 3.2.2. Sixth Graders

Consistent with children from second grade, own school (35.2%), home (35.2%), and streets (14.3%) were the most visited settings among sixth-grade children on weekdays, and home (54.5%), streets (17.2%), and other places (15.0%) on weekend days, respectively (Table 2). Furthermore, this age group also recorded significantly more minutes of total matched wear time on weekdays compared to weekend days (+75.6 min, *p* = 0.003). Within the different settings, sixth graders spent significantly more time on weekdays at their own school (+226.7 min, *p* < 0.001) and in recreational facilities (+2.9 min, *p* = 0.022), while significantly more time was accumulated at home (+76.8 min, *p* < 0.001) and in other places (+18.8 min, *p* < 0.001) on weekend days.

### 3.3. Daily Minutes of MVPA Spent in Settings

#### 3.3.1. Second Graders

Table 3 provides results on the median daily minutes of MVPA spent in different settings separated for weekdays and weekend days. In total, second-grade children spent a median time of 68.9 min in MVPA on a regular school day, whereby most of their MVPA was accumulated at own school (40.0%), on streets (21.1%) and at home (13.3%). On weekend days, second graders accumulated a total median of 59.6 min in MVPA, whereof 21.4% was spent on streets, 21.3% at home and 17.8% in other places. Significantly more MVPA on weekdays was recorded at own school (+24.6 min, *p* < 0.001), at other school (+2.6 min, *p* < 0.001) and on streets (+2.7 min, *p* < 0.001), whereas on weekend days, a significantly greater amount of MVPA was accumulated at home (+3.1 min, *p* < 0.001), in recreational facilities (+0.1 min, *p* < 0.001) and outside the city (+0.1 min, *p* < 0.001).

#### 3.3.2. Sixth Graders

Sixth graders accumulated the majority of their MVPA on weekdays at own school (33.9%), on streets (33.8%) and at home (11.9%), and during weekends on streets (32.0%), at home (22.4%) and in other places (16.6%), respectively (Table 3). Sixth-grade children achieved a significantly higher amount of MVPA on weekdays than they did on weekend days (+25.5 min, *p* < 0.001). Within settings, significant differences with children spending more minutes in MVPA during the school week than on weekend days were only found at own school (+17.1 min, *p* < 0.001) and on streets (+8.1 min, *p* < 0.001).

### 3.4. Proportion of Time Spent in MVPA in Settings

#### 3.4.1. Second Graders

Table 4 provides results on the proportion of time spent in MVPA in total and across settings by week and weekend days. To indicate how many children visited the correspondent setting, the number of children (*n*) is presented. Overall, the second-grade children recorded 11.3% of their total time in MVPA on weekdays and 10.8% on weekend days, respectively (*p* > 0.05). On weekdays, the highest proportions of time spent in MVPA were observed on streets (17.6%), in recreational facilities (17.4%) and at other schools (17.2%), while on weekend days, children were most active at own school (39.4%), in recreational facilities (16.1%) and at other schools (12.6%). Significantly higher proportions on weekdays compared to weekend days were found at other schools (+4.6%, *p* = 0.025), on streets (+6.9%, *p* < 0.001) and in other places (+1.9%, *p* = 0.043). In contrast, own school was the only setting in which second-grade children recorded a considerably higher proportion of time spent in MVPA on weekend days (+29.4%, *p* < 0.001). Both on week and weekend days, the smallest proportion of time spent in MVPA was observed in the home environment (6.0% and 5.5%, respectively).

#### 3.4.2. Sixth Graders

Among sixth graders, the proportion of time spent in MVPA on weekdays were largest at other school (21.9%), on streets (20.5%), and in recreational facilities (19.8%), whereas the highest relative activity level on weekend days was observed at own school (16.3%), at other school (13.6%), and on streets (9.7%). In contrast to second graders, sixth-grade children showed a significantly more active behaviour on weekdays compared to weekend days (+3.3%, *p* < 0.001). Accordingly, they recorded significantly higher MVPA-proportions on weekdays compared to weekends at other schools (+8.3%, *p* < 0.001), in recreational facilities (+10.6%, *p* = 0.003), on streets (+10.8%, *p* < 0.001), and in other places (+2.3%, *p* < 0.001). A significantly more active behaviour on weekend days was only found outside the city (+0.2%, *p* < 0.035). Consistent with second graders, the smallest proportion of time spent in MVPA on weekdays as well as on weekend days was found at home in this age group (3.0% and 2.6%, respectively).

## 4. Discussion

The aim of the present study was to investigate differences in the spatial PA patterns between weekdays and weekends in second and sixth-grade children living in two urban areas in Switzerland. In both age groups, we found several differences between week and weekend days in relation to the total time spent as well as to the absolute and relative level of PA in total and within various settings. While second graders did not show a significant difference in their absolute or relative PA level between week and weekend days over all settings, sixth-grade children were significantly less active on weekend days. On weekdays, the school environment was the most frequently used setting in which both age groups accumulated more than one third of their total MVPA. Although significantly less time was spent at their own school on weekend days, the proportion of time spent in MVPA was much higher during this time of the week compared to school days, especially among second-grade children. In contrast, second and sixth graders spent most of their weekend time at home where they engaged in significantly more MVPA on weekend days compared to weekdays. Nevertheless, the proportion of time spent in MVPA at home did not differ significantly between time segments and was generally low in both age groups. Furthermore, we found a significantly higher PA level in the street environment on weekdays compared to weekend days in both age groups, although second and sixth graders spent a similar amount of time there on week and weekend days.

### 4.1. Differences in General Physical Activity Patterns between Weekdays and Weekends

Both second and sixth graders recorded significantly more minutes of matched wear time data on weekdays compared to weekend days. However, while sixth-grade children also engaged in significantly more MVPA and showed a higher proportion of time spent in MVPA during the school week compared to weekend days, second graders did not show a significant difference between time segments in relation to their absolute or relative PA level over all settings. A study by Trost and colleagues showed that younger children exhibited higher levels of PA on weekends, whereas older children and adolescents were more active on weekdays relative to weekends [51]. This is somewhat consistent with the findings in the present study. Moreover, previous studies further reported that the decline in MVPA with age is consistently higher on weekends than on weekdays [14,16]. Although we did not statistically prove differences between age groups, this finding is approximately congruent with our study results: second graders were engaging in more MVPA than sixth graders on both week and weekend days, whereby this difference was particularly accentuated on weekend days. Thus, in line with previous research [12], our findings support the promotion of weekend PA, especially when children grow older.

### 4.2. Differences in Context-Specific Physical Activity Patterns between Weekdays and Weekends

When focusing on the various settings, the school environment was the most used setting on weekdays in both age groups and therefore significantly more time in MVPA was then accumulated within this setting compared to weekend days. This finding is in line with other studies which already reported that children spent the majority of time on weekdays at school and that this environment is an important setting regarding the daily accumulation of MVPA [32,47,52]. However, only a few studies at present have investigated how this environment is used by children outside scheduled school hours during weekdays and, especially, during weekend days. Klinker and colleagues [32] found that school grounds are important places to spend time on weekdays during after school hours but less so on weekend days. Only 34.5% of the participants used a school ground during weekends and the median time they spent at school was zero minutes [32]. In the present study, slightly more children (47.3% second graders and 43.9% sixth graders, respectively) used either the school grounds of their own school or those of another school on a weekend day, but the median time spent in both settings was also zero minutes among all participants. Despite this low use of school grounds during weekends, the high MVPA-proportion recorded by children who spent time there during this time segment may still indicate the potential importance of the school setting as an activity-promoting environment. Accordingly, both age groups also recorded noticeably high MVPA-proportions when spending time at other schools. These results confirm a previous study that indicated that an open and unrestricted access to school grounds during leisure time for organized sports and unstructured play is an urgent need [32]. However, further studies should gain more knowledge on what children are doing when staying on school grounds. It can be hypothesized that one reason for the high relative PA level is that the school infrastructure is often used for organized PA in the context of sports clubs as well as voluntary sports at school. To conclude, findings in the present study potentially suggest that targeting a broader use of school grounds during non-school hours on week and weekend days for both organized and unstructured PA could be a promising avenue for future interventions.

The home environment was the only setting in which children from both age groups accumulated significantly more time in MVPA on weekend days compared to weekdays or showed a trend to do so. The higher absolute amount of MVPA recorded on weekend days can mostly be attributed to the more frequent use of this setting during this part of the week. It is hardly surprising that children are spending more time at home when the structured school routine is absent, which probably leads to more opportunities to accumulate health-enhancing PA in this setting. However, when considering the relative MVPA outcome, a similar and particularly small MVPA-proportion was recorded at home on week and weekend days and across both age groups. This finding supports previous research that identified home as an environment in which children are less active than when they are out of it [53,54]. The increasing range of screen-based technologies has led to a higher engagement in sedentary activities such as TV viewing, playing computer games, or surfing the internet and may attract children to stay at home rather than using outdoor spaces which are more conducive for PA [53,54,55]. Therefore, interventions which encourage children to go out more for walking and playing outdoors, especially on weekends when they have a greater choice in how they spend their time, would be an important approach to promote PA among primary school children, particularly the older ones.

Although both age groups spent a similar amount of time during week and weekend days on streets, they showed a significantly higher absolute and relative level of PA in this setting on weekdays compared to weekend days. This different behaviour in the street environment can partly be explained by the absence of ways to and from schools during non-school days. Previous studies showed the significant contribution of the journey to school to total PA [46,56,57] and that children who walk or cycle to school have a greater overall PA level than those who are driven to school or take public transportation [58]. Furthermore, given that peer support is particularly relevant on school days [59], children probably are more likely to use the street environment for informal PA with their friends such as playing tag, skipping, or ball games on weekdays than on weekend days. In contrast, the higher time spent in motorised transport may be a reason for the more sedentary behaviour within this setting during non-school days [60]. Accordingly, the promotion of active commuting to other destinations than school could contribute toward achieving higher PA levels [61], which would be of particular value for weekend days in both age groups. To reach this goal, safe residential streets and neighbourhoods are required, especially as the lack of perceived neighbourhood safety seems to be associated with lower levels of active transport [62,63].

Other places were significantly more visited by both age groups on weekend days than on weekdays, which is obvious when time-consuming school-related activities are absent. However, second as well as sixth graders showed a significantly higher relative PA level when visiting this setting during school days. Similar to the street environment, the more pronounced peer influence during weekdays may be responsible for this difference [59]. It can be hypothesized that during weekdays, other places such as the near neighbourhood, private gardens, or other’s home are rather visited in the company of peers or close friends engaging in active free-play. Appropriately, different studies could show that presence of peers and friends is associated with higher activity levels [64,65]. In contrast, during weekend days, children are more likely to use this setting together with family members engaging in more sedentary activities. Dunton and colleagues [66] found that although most of the parent-child pairs performed some joint MVPA, the duration of these activities was short. Moreover, this low activity behaviour was particularly accentuated in parents with older children as they spent more time in sedentary activities and less time in PA together [66]. The same authors therefore concluded that the promotion of joint parent-child PA in families with older children may be a promising method to combat the age-related decline in PA [66]. In accordance with this conclusion, our results further indicate that the promotion of joint parent-child PA would predominantly be useful during weekend days.

The use of recreational facilities such as parks, playgrounds, and sport facilities was generally low during week and weekend days and among both age groups. This result is in line with previous research reporting that the use of green space as an absolute measure of time is low [28,46]. In addition to other studies, we could further observe a tendency that second graders compared to sixth graders spent more time in recreational facilities, which was particularly true for weekend days. Second-grade children further achieved significantly more minutes of MVPA on weekend days compared to weekdays in this setting, while sixth graders were significantly less active in parks and sport facilities during weekends. In line with a recent study reporting that open spaces and parks offer a common setting for joint parent-child PA [67], we can consider that second-grade children rather tended to visit parks and sport facilities accompanied by their parents and were engaging together in PA. Furthermore, the sixth graders’ low use of parks and sport facilities can partly be explained by the fact that this setting is more likely to be visited in summer months than during winter and early spring, which is when the study of the sixth graders was conducted [44]. Nevertheless, the high proportion of time spent in MVPA recorded in both age groups confirms previous research that already established that green space is highly conducive for PA even though rarely visited [28,46]. Therefore, future interventions should target the more frequent use of recreational facilities, especially in older children and during weekend days. This also includes the promotion of organized PA in the context of sports clubs or commercial sports providers as the benefits of structured activities have been well proven and joining organized sports during childhood appears to increase the chance for an active lifestyle in young adulthood [68,69].

### 4.3. Limitations

The present study has several limitations. We assessed the PA patterns of a convenience sample of second and sixth graders from two urban regions and surveyed them during early spring and in summer months, respectively. Therefore, findings are not generalizable to other populations and seasons. Given that the participation was voluntary for every pupil in a participating class, we cannot exclude certain effects due to selection bias. As both the city and season differed between second and sixth graders for administrative reasons, we decided not to investigate differences between age groups, although this comparison would represent an important issue and should be considered in future research. The combination of accelerometry and GPS is a promising method to gain further insights in children’s PA patterns and studies using this approach are increasing in number. However, the use of GPS and accelerometry is associated with several problems. The accuracy of the GPS signal varies with the environment. Urban canyons, vegetative cover, or indoors within structures impermeable to satellite signals are associated with inaccurate and missing GPS positions and therefore can lead to misclassification errors and under- or overrepresentations of certain settings [45]. Although we did not impute missing data, we tried to reduce issues with spatial inaccuracy by identifying invalid GPS points and choosing buffer zones around polygons. However, misclassification bias remains possible. Moreover, accelerometer-based measurements of PA also have limitations, predominantly consisting of the inability to assess activities accurately in which the body’s centre of gravity is relatively fixed such as cycling [70], the absence of a universal consensus on processing methods and accelerometer cut points for determining MVPA [71], and reactivity issues [72]. The inclusion criterion of at least one valid week and weekend day of four full hours of combined data was chosen to maximize data retention for analysis. This criterion is rather low in comparison with other studies [32,41], and might limit the reliability of the presented results. However, with a daily median of more than nine hours of combined data in both age groups, we still reached a satisfying amount of data that enabled us to conduct a full day pattern analysis.

## 5. Conclusions

To the best of our knowledge this is the first study which objectively assessed by means of accelerometry and GPS and by using both an absolute and relative outcome measure of MVPA the spatial PA behaviour in primary school children and investigated whether there are differences between week and weekend days in two different age groups. In both second and sixth-grade students we could find several differences in the spatial PA patterns between week and weekend days. These differences emphasise the need of targeting weekend PA for future interventions, especially when children grow older. Future interventions should encourage children to use outdoor spaces, such as parks and sport facilities, more frequently on weekends rather than stay at home and should also encourage children to commute actively on weekend days in the absence of the daily journey to school. Further studies need to confirm whether the present findings also apply in rural and suburban populations and in different seasons of the year. Furthermore, future cross-sectional and longitudinal studies should investigate age differences in the spatial PA behaviour between week and weekend days to shed light on the age-related decline in PA when children grow older.

## Figures and Tables

**Table 1:** General characteristics of the study population, stratified by age.

Characteristic	Second Graders	Sixth Graders	*p*-Value
Age in years, mean (SD)	8.5 (0.3)	12.5 (0.4)	<0.001 **
Gender, boys, *n* (%)	38 (51.4)	45 (45.9)	0.480 *
Gender, girls, *n* (%)	36 (48.6)	53 (54.1)	–
Body height in cm, mean (SD)	133.4 (5.6)	154.7 (7.7) ^1^	<0.001 **
Body weight in kg, median (IQR)	28.4 (25.6–32.1)	44.2 (38.2–53.8) ^1^	<0.001 ***
BMI in kg/m^−2^, median (IQR)	15.9 (15.1–17.5)	18.3 (16.5–20.7) ^1^	<0.001 ***
Daily wear time in min, median (IQR)	754.6 (702.6–799.5)	772.4 (727.1–810.4)	0.029 ***
Daily combined data in min, median (IQR)	575.4 (493.4–621.6)	579.0 (498.6–659.7)	0.435 ***
Availability of GPS data in %, median (IQR)	76.8 (68.6–84.6)	78.1 (68.1–86.8)	0.569 ***
Wear days, median (IQR)	7.0 (7.0–7.0)	7.0 (7.0–7.0)	0.264 ***
Valid weekdays, median (IQR)	5.0 (5.0–5.0)	5.0 (4.0–5.0)	0.301 ***
Valid weekend days, median (IQR)	2.0 (2.0–2.0)	2.0 (2.0–2.0)	0.031 ***

Bold: significant difference at *p* < 0.05; ^1^ Missing values from 5 participants; * χ^2^-test; ** *t*-test; *** Mann-Whitney-*U*-test; SD = Standard Deviation; BMI = Body Mass Index; IQR = Interquartile Range.

**Table 2:** Daily minutes of total time spent in settings, by weekday and weekend day, stratified by age.

Setting	Weekday			Weekend Day			*p*-Value *
	*n*	Median	IQR	*n*	Median	IQR	
**Second Graders**							
Total	74	597.2	540.2–668.0	74	554.3	464.2–604.2	**0.047**
Home	74	134.0	71.4–171.9	74	172.3	85.4–302.3	**<0.001**
Own school	74	244.6	204.7–285.0	74	0.0	0.0–0.0	**<0.001**
Other school	74	19.6	4.6–31.8	74	0.0	0.0–4.8	**0.020**
Recreation	74	14.5	3.4–37.1	74	18.4	0.0–71.8	**<0.001**
Street	74	83.6	63.1–116.3	74	92.4	60.4–137.1	0.234
Other	74	58.5	41.9–79.0	74	65.9	26.9–121.7	**0.032**
Outside	74	0.0	0.0–2.4	74	5.7	0.0–97.8	**<0.001**
**Sixth Graders**							
Total	98	610.6	551.2–695.9	98	535.0	479.0–622.6	**0.003**
Home	98	216.3	169.4–283.4	98	293.1	164.1–412.8	**<0.001**
Own school	98	226.7	152.9–283.1	98	0.0	0.0–0.0	**<0.001**
Other school	98	1.6	0.0–27.9	98	0.0	0.0–2.6	0.355
Recreation	98	2.9	0.0–18.2	98	0.0	0.0–7.1	**0.022**
Street	98	78.0	62.8–105.9	98	81.2	47.2–121.0	0.490
Other	98	51.5	37.2–82.0	98	70.3	40.1–99.2	**<0.001**
Outside	98	0.0	0.0–0.0	98	0.0	0.0–51.8	0.052

Bold: significant difference at *p* < 0.05; * *p*-values are calculated from F-test based on Sattethwaite’s or Kenward-Roger approximation; IQR = Interquartile Range.

**Table 3:** Daily minutes of MVPA in settings, by weekday and weekend day, stratified by age.

Setting	Weekday			Weekend Day			*p*-Value *
	*n*	Median	IQR	*n*	Median	IQR	
**Second Graders**							
Total	74	68.9	53.6–83.1	74	59.6	40.3–82.4	0.313
Home	74	7.7	4.4–12.3	74	10.8	4.2–21.8	**<0.001**
Own school	74	24.6	17.3–37.8	74	0.0	0.0–0.3	**<0.001**
Other school	74	2.6	0.8–5.0	74	0.0	0.0–0.5	**<0.001**
Recreation	74	2.5	0.6–7.4	74	2.6	0.0–13.3	**<0.001**
Street	74	13.6	9.6–18.7	74	10.9	3.7–18.3	**<0.001**
Other	74	6.1	3.4–9.4	74	7.5	1.9–15.8	0.573
Outside	74	0.0	0.0–0.1	74	0.1	0.0–11.5	**<0.001**
**Sixth Graders**							
Total	98	52.7	44.6–65.1	98	27.2	18.2–48.2	**<0.001**
Home	98	5.8	4.0–9.2	98	6.4	4.5–11.2	0.063
Own school	98	17.1	13.7–23.1	98	0.0	0.0–0.0	**<0.001**
Other school	98	0.3	0.0–5.2	98	0.0	0.0–0.1	0.482
Recreation	98	0.4	0.0–3.0	98	0.1	0.0–1.2	0.812
Street	98	16.4	12.2–23.3	98	8.3	2.4–16.0	**<0.001**
Other	98	4.1	2.2–5.8	98	3.5	1.6–6.5	0.977
Outside	98	0.0	0.0–0.0	98	0.0	0.0–1.5	0.825

Bold: significant difference at *p* < 0.05; * *p*-values are calculated from F-test based on Sattethwaite’s or Kenward-Roger approximation; IQR = Interquartile Range; MVPA = Moderate to Vigorous Physical Activity.

**Table 4:** Proportion of time spent in MVPA in settings based on children who visited the corresponding setting (*n*), by weekday and weekend day, stratified by age.

Setting	Weekday			Weekend Day			*p*-Value *
	*n*	Median	IQR	*n*	Median	IQR	
**Second Graders**							
Total	74	11.3	9.1–13.6	74	10.8	7.5–15.5	0.852
Home	74	6.0	4.7–9.1	69	5.5	4.1–8.8	0.438
Own school	74	10.0	8.2–13.2	19	39.4	17.6–48.3	**<0.001**
Other school	64	17.2	10.2–23.8	29	12.6	5.7–24.3	**0.025**
Recreation	67	17.4	9.3–27.7	56	16.1	7.2–27.0	0.272
Street	74	17.6	11.9–22.7	73	10.7	7.5–15.8	**<0.001**
Other	74	11.2	7.3–14.7	72	9.3	6.4–15.5	**0.043**
Outside	24	8.6	3.4–12.6	37	8.4	2.8–16.3	0.871
**Sixth Graders**							
Total	98	8.6	7.2–10.4	98	5.3	3.4–9.4	**<0.001**
Home	98	3.0	2.1–4.1	98	2.6	1.6–4.4	0.747
Own school	98	8.8	5.9–11.7	21	16.3	8.0–38.7	0.437
Other school	65	21.9	9.5–30.0	32	13.6	4.5–35.3	**<0.001**
Recreation	70	19.8	8.5–36.3	54	9.2	3.3–30.8	**0.003**
Street	98	20.5	14.0–28.9	97	9.7	3.4–16.6	**<0.001**
Other	98	7.4	4.8–10.1	96	5.1	2.6–8.8	**<0.001**
Outside	17	5.1	2.8–11.8	31	5.3	1.9–12.3	**0.035**

Bold: significant difference at *p* < 0.05; * *p*-values are calculated from F-test based on Sattethwaite’s or Kenward-Roger approximation; IQR = Interquartile Range; MVPA = Moderate to Vigorous Physical Activity.
